# Up-regulation of CREB-1 regulates tendon adhesion in the injury tendon healing through the CREB-1/TGF-β3 signaling pathway

**DOI:** 10.1186/s12891-023-06425-7

**Published:** 2023-04-25

**Authors:** Li-Ming Wu, Yun-Jiao Wang, Shuai-Feng Li, Jing-Kun Wang, Jun Liu, Chao-Chao Fan, Yan Xiong

**Affiliations:** 1 Department of Orthopaedics, Daping Hospital, Army Medical University, 10 Changjiang Branch Road, Yuzhong District, Chongqing, 400042 People’s Republic of China; 2 Department of Orthopaedics, Chongqing Public Health Medical Center, Chongqing, 400030 People’s Republic of China; 3 Department of Sports Medicine Center, State Key Laboratory of Trauma, Burn and Combined Injury, Southwest Hospital, Army Medical University, Chongqing, 400038 People’s Republic of China; 4 Department of Spinal Surgery, the General Hospital of the People’s Liberation Army Tibet Military Area Command, Lhasa, 850007 People’s Republic of China; 5 Department of Orthopaedics, the People’s Hospital of Nanchuan, Chongqing, 408400 People’s Republic of China

**Keywords:** Tendon_1_, Adhesion_2_, Healing_3_, TGF-β3_4_, CREB-1_5_

## Abstract

**Aim:**

To explore the mechanism of the healing of tendon tissue and anti-adhesion, and to discuss the role of the transforming growth factor-β3 (TGF-β3)/cAMP response element binding protein-1 (CREB-1) signaling pathway in the healing process of tendons.

**Method:**

All mice were divided into four groups of 1, 2, 4, and 8 weeks respectively. Each time group was divided into four treatment groups: the amplification group, the inhibition group, the negative group, and the control group. When the tendon injury model was established, the CREB-1 virus was injected into the tendon injury parts. A series of methods such as gait behaviourism, anatomy, histological examination, immunohistochemical examination and collagen staining were employed to assess the tendon healing and the protein expression of TGF-β3, CREB-1, Smad3/7 and type I/III collagen (COL-I/III). CREB-1 virus was sent to tendon stem cells to assess the protein expression of TGF-β1, TGF-β3, CREB-1, COL-I/III by methods such as immunohistochemistry and Western blot.

**Results:**

The amplification group showed better gait behaviourism than the inhibition group in the healing process. The amplification group also had less adhesion than the negative group. Hematoxylin–eosin (HE) staining of tendon tissue sections showed that the number of fibroblasts in the amplification group was less than the inhibition group, and the immunohistochemical results indicated that the expression of TGF-β3, CREB-1, and Smad7 at each time point was higher than the inhibition group. The expression of COL-I/III and Smad3 in the amplification group was lower than the inhibition group at all time points. The collagen staining indicated that the ratio of type I/III collagen in the amplification group was higher than the negative group at 2,4,8 week. The CREB-1 amplification virus could promote the protein expression of TGF-β3, CREB-1 and inhibit the protein expression of TGF-β1 and COL-I/III in the tendon stem cells.

**Conclusion:**

In the process of tendon injury healing, CREB-1 could promote the secretion of TGF-β3, so as to promote the tendon healing and have the effect of anti-adhesion in tendons. It might provide new intervention targets for anti-adhesion treatment of tendon injuries.

**Supplementary Information:**

The online version contains supplementary material available at 10.1186/s12891-023-06425-7.

## Introduction

As the dense connective tissue at the end of the muscle, the tendon plays the role of connecting the bones and muscles. When the muscle contracts, the tendon can transmit the strength to the bone and make the joint move [1]. Tendons play an important role in the movement of the body. Tendon injuries are one of the most common sports injuries, especially for athletes and soldiers. The long-term chronic tendon injury can cause tendinopathy. It can be gradually improved through rest and physical therapy [2]. But the acute injury is mainly manifested as a tendon tear or rupture, which usually needs surgical treatment. The operation is very mature and has achieved good clinical effects through continuously improving the operation methods to reduce the surrounding injury, using biological and physical barriers during the operation and physical rehabilitation treatment after the operation to reduce adhesion around the tendon [3–5]. The tendons are characterised by oligo-cytosis, oligo-vascularity, and oligo-innervation, so it is difficult to heal after injury. Although a lot of research has been done on the repair mechanism of tendon injuries, the adhesion of the tendon and surrounding tissue after operation is still a severe problem in clinical work. If the adhesion of the tendon can be reduced in the healing process, the outcome of the treatment may further improve, but the mechanism of tendon healing remains unclearly and incompletely described. Based on this, further research on tendon injury repair mechanisms can provide new treatment targets for reducing tissue adhesion after tendon injury.

During the tissue repair process, a large number of growth factors and inflammatory factors are involved. TGF-β, CREB, and other factors have been found to play an important role in the process of tendon injuries [6, 7]. TGF-β is a multifunctional family cytokine that plays an important role in cell proliferation, differentiation, injury repair, and fibrosis. TGF-1 and TGF-3 isomers are the subject of much more research. Furthermore, early studies reported TGF-β can regulate scar formation in the process of tissue healing, whereas TGF-β1 and TGF-β3 play the opposite role [8, 9], and it was also found that TGF-β is active in the process of tendon healing in the study of tendon [10] and has a close relationship with fibrosis. CREB, as a transcription factor, can mediate a variety of physiological signals in cells [11], allowing it to participate in a variety of cell reaction processes. Of course, it can also regulate tissue fibrosis [12], and research on tendon tissue has revealed that the inflammation response of the tendon and CREB is closely linked [13]. TGF-β and CREB both play roles in the regulation of the inflammation during the tendon healing [14]. Our preliminary work has proved that there is a close connection between them [15], but what correlation they have during the healing process needs further research. As a result, this study performed a mechanism analysis around TGF-β to clarify the effects of related proteins on tendon adhesion during tendon healing.

## Materials and methods

### Experimental materials

#### Animal

All the healthy c57/bl6 male mice (age: 6-8 weeks; weight: 15-20g) were from the Daping Hospital Animal Experiment Centre of the Army Medical University. They could get food and water freely, and experiments took place in the animal room under standard conditions. Experimental animals were not used for reproduction. 80 mice were used in this study. There were 5 mice in each treatment group at each time point.

#### Main instruments and reagents

Catwalk XT platform (Noldus Information Technology, Netherlands), high-speed frozen centrifugal machines (Kejun Instrument Company, USA), Nanodrop UV spectrophotometer (Thermofisher, USA), fluorescence quantitative PCR (PALL, 65421), paraffin wheel rotor (Leica, Germany), Sodium pentobarbital, RNA extract kit (Sangon Biotech, Shanghai, B618133-0050), cDNA synthetic kit (Sangon Biotech, Shanghai, B532445-0020), qPCR reaction Mix (Sangon Biotech, Shanghai, B110031-0001), immunohistochemical kit (Sangon Biotech, Shanghai, E607250-0100), Anti-TGF-β1 antibody (Abcam ab92486), Anti-TGF-β3 antibody (Abcam ab15537), Anti-CREB antibody (Abcam ab31387), Anti-Smad3 antibody (Abcam ab208182), Anti-Smad7 antibody (Abcam ab216428), Anti-Collagen I antibody (Abcam ab270993), Anti-Collagen III antibody (Abcam ab7778), LV-Creb1 (Genechem, 43415–1), LV-Creb-RNAi (Genechem, 70035–1), negative control virus (Genechem, CON335).

### Methods

#### Animal model establishment

All the experimental mice were subjected to the flexion tendon injury model on all their rear limbs. Sodium pentobarbital were injected into their abdominal cavities. After intraperitoneal anesthesia, shaving and skin preparation are performed on the palms of both hind limbs. After disinfection, slice the skin along the long axis. When the skin and subcutaneous tissues were sliced, the flaps were held on both sides to expose the flexion tendon, which was to be severed by a blade at the midpoint. A 5–0 absorbable suture was used to repair the tendon using the improved Kessler suture method. The CREB-1 virus was injected at a distance of 5 mm from the suture, and the amplification virus was injected for the amplification group, the inhibition virus for the inhibition group, and the negative virus for the negative group. The control group would not be injected. A 2–0 absorbable suture was used to simply and intermittently suture the skin. The operated limbs were not specially fixed, and the mice went on to be raised under the same conditions in single cages. All mice can get early mobilization, enough water and food freely The experimental materials were collected from the hind feet of the experimental animals from the groups in the 1st, 2nd, 4th and 8th weeks after the surgery. After anesthesia, the skin and the subcutaneous tissues were cut along the original surgical incision. The surrounding adhering and free healing tendon tissue were separated. The complete tendon specimens were collected, in total 1 cm, from the distal and proximal ends of the suture. The animals should be raised normally after suturing the wound. Each group should obtain 40 tendon specimens: 10 from the amplification groups, 10 from the inhibition groups, 10 from the negative groups, and 10 from the control groups.

#### Gait analysis

On the day of sampling in each experimental group, the Catwalk platform was used to observe the animals’ gait, and the data collected by the platform were to be analyzed. It mainly analyzed and compared the step cycle and average speed indicators. The cycle (s) is the time between the first contact of the glass plate and the subsequent one by the same claw. The average speed (cm/s) is the speed at which the experimental object walked across the glass plate. The step cycle and average speed are parameters related to mobility analysis. A short step cycle and a high average speed indicate that the animal’s movement function is good.

#### Gross observation

When sampling the tendon at each time point, the healing of the exposed tendon was observed through an optical microscope and photos were taken for the record. According to the healing of the tendon, the Tang’s grade method was used to grade the tendon specimens [16]: Grade I: There is no adhesion around the tendon, which shows granulation tissue. Grade II: There is a small amount of membrane adhesion at the suture of the tendon, with granulation tissue, and the tendon slip is slightly limited. Grade III: There is a small loose adhesive band that is easy to separate from the surface of the tendon, and the tendon slip is partial limited. Grade IV: There is moderately adhesion, the tendon has a certain degree of activity, and the slip is obviously limited. Grade V: There were severe extensive adhesion, poor mobility and no boundary between the tendon and the peritendinous tissue. The grade of tendon healing was compared and analyzed.

#### Histological observation

The obtained tendon specimens were adequately fixed and embedded, and they were cut into a paraffin thickness of 6 μm. They were stained with HE and examined under a microscope to determine the number of cells on the broken end of the tendon and the collagen arrangement. Pictures were taken under the 100-fold optical microscope. Each slice selected 6 good visual fields randomly. Image-Pro Plus 6.0 was used to count the number of cells in each visual field and to analyse the differences between the groups.

#### Immunohistochemical observation

Three sections of paraffin-embedded tendon tissues will be stained with immunohistochemistry.. Methods: dewaxing in water after baking slices, boiling to repair antigen, blocking by 3%H_2_O_2_. TGF-3, CREB, Smad3/7, and COL-I/III antibodies were incubated overnight at 4 °C After incubation, add the IgG of biotin labeled goat anti-rabbit. DAB was used to develop color. Hematoxylin solutions were used for re-staining. 6 well-stained visual fields were selected from each section. Photos were taken under a 100-fold optical microscope. The expression of TGF-β3, CREB, Smad3/7, COL-I/III was locationally and qualitatively analysed. Image-Pro Plus 6.0 was used for quantitative analysis.

#### Collagen staining observation

Three sections of the paraffin-embedded tendon tissues were used to be stained Sirius-red. Methods: After drying, the sections were dewaxed to induce dehydration. Harris hematoxylin solutions were used for staining. Picric acid and Sirius-red solutions were also used for staining. Water-free ethanol was used for direct color division and dehydration. The observation was conducted by optical microscope. The xylene was seen as transparent. It was air dried in the fume hood before being sealed with neuter gum. A microscopic examination was to be conducted. A polariscope (100-fold) was used to observe 4 types of collagen fibers: Type I collagen fiber: tightly arranged, showing strong birefringence, and are yellow or red. Type II collagen fiber was loosely knitted netlike and showed weak birefringence in a variety of colors. Type III collagen fiber: showed weak birefringence, with green fine fibers. Type IV collagen fiber: showing weak birefringent basement membranes, which were pale yellow.

#### Tendon stem cell culture identification

**Fig. 1:**
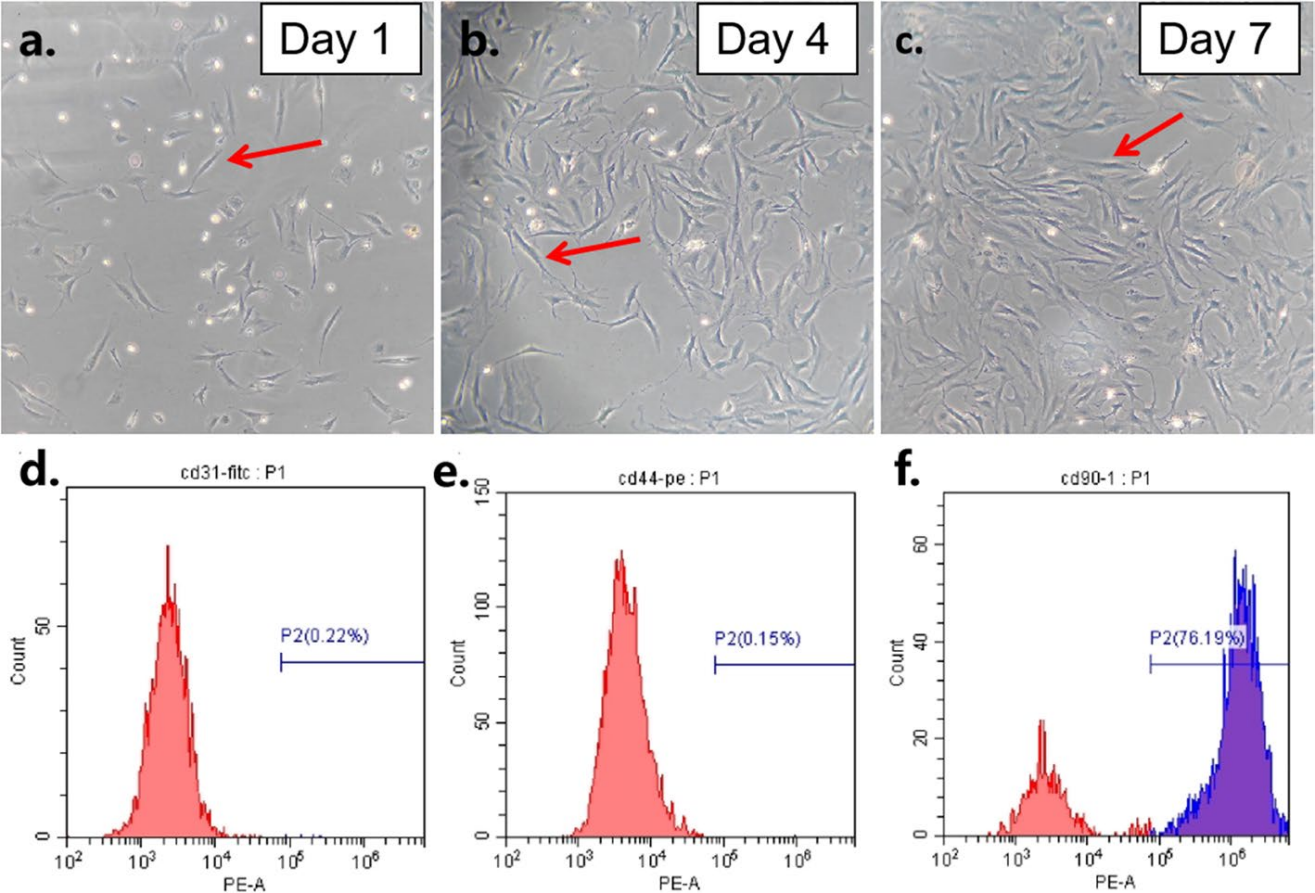
The culture and identification of tendon stem cells: **a** Tendon stem cells P2, day 1. **b** Tendon stem cell P2, day 4. **c** Tendon stem cell P2, day 7. The red arrow points to the tendon stem cells. **d**, **e**, **f** The flow cytology detection result of Tendon stem cell P2

The tendon stem cells were extracted in accordance with our previous experimental method [17]. After exercising euthanasia on the mice, the Achilles tendon and flexion tendon of both rear feet were dissected. Only the intermediate tissues were collected. And the connective tissues around the tendon were carefully resected. The intermediate tissues were chopped in aseptic PBS, digested for 2.5 h at 37 ℃ by the 3 mg/ml type I colloidase (Sangon Biotech, Shanghai, NO. A004214), and 70 mm cell filters were used to create a unicellular suspension (Becton Dickinson, Franklin Lakes, NJ, USA). The cells were washed with PBS, 300G centrifugal for 5 min, and the cells above the culture were resuspended in 10% foetal bovine serum (BI, 04–002-1ACS), 100U/ml penicillin, 100 mg/ml chaincin (Sangon Biotech, Shanghai, NO. B540732), and 2 mmol/L low sugar medium (Gibco, Carlsbad, CA, USA). The diluted cells were planted on the cell culture vessels respectively. They were to be cultured at 37 ℃ in a 5% carbon dioxide incubator for 2 days, and then they were washed twice with PBS to remove the non-adherent cells. On the seventh day of cultivation, they were digested with trypsase (Hyclone, SH30042), and mixed together as the "generation 0 " of the cell to be cultured. The tendon stem cells were cultured to second generation(P2), it can be observed that the tendon stem cells are shuttle-shaped and grow evenly on the culture vessel through the optical microscope. The P2 cells were used for subsequent experiments. The tendon stem cells specificity marking antibody of CD90(Thermofisher, 12–0902-82) is used for identifying TSC by flow cytology. The results indicated that the expression of CD90 is positive, and CD31 is not. It complied with the specific expression of the tendon stem cell antigen and could be used for further cell experiments (Fig. 1).

#### Lentivirus transfection

Tendon stem cells were inoculated into each well of 6-well culture plates at 3–5 × 10^4^ cells/ ml in complete culture medium. In order to infect cells, add 40UL virus at a concentration of 1 × 10^8^ to the culture vessel with an MOI of 20. The amplification group added the amplified virus, inhibition group added the inhibition virus, negative group added the negative virus, the control group added the same amount of culture medium, and then, added 960 μl of virus transfection liquid. After blowing evenly, they were incubated for 16 h at 37℃ in a 5% carbon dioxide incubator. After 16 h, replace the complete culture medium and continue to culture for another 72 h. 72 h later, the transfection efficiency was to be observed by fluorescence microscopy. If the area of green fluorescence is greater than 80%, the transfection is successful.

#### Cellular immunohistochemistry

The successfully transfected cells used 96-well plate for climbing. 3 days later, the cells were subjected to immunohistochemistry. At first, cells were fixed with 4% paraformaldehyde for 30 min, and then, they were incubated at room temperature with 3% triton for 30 min and blocked with 5% goat serum at room temperature for 30 min. Antibody (Anti-TGF-β3 antibody, Anti-CREB antibody, Anti-Smad3 antibody, Anti-Smad7 antibody) incubated the treated cells overnight at 4 °C., Anti-Collagen I antibody, Anti-Collagen III antibody). The IgG of biotin labeled goat anti-rabbit was added and incubated 30 min. DAB was used to develop color and hematoxylin was used to dye again. Photos were taken under a 400-flod optical microscope. The expression of the TGF-β3, CREB, Smad3/7, COL-I/III was locationally and qualitatively analyzed.

#### RNA extraction and real-time RT-qPCR

The transfected cells were to be detected by real-time quantitative fluorescence PCR. Total RNA was to be extracted by the Trizol method, and cDNA was synthesized by reverse transcription reaction. The total volume of the reaction system was 20 μl. Real-time fluorescent quantitative PCR used cDNA, SYBR Green Master Mix (VAZYME) and TGF-β 3, CREB gene specific primers. The reaction conditions were: 1 cycle at 95℃ for 10 min, 40 cycles at 95℃ for 15 s, 40 cycles at 60℃ for 60 s. 1 cycle at 95℃ for 15 s, 1 cycle at 60 °C for 60 s, and 1 cycle at 95℃ for 15 s. After the reaction, the Ct value of each sample was automatically analyzed by a computer. The relative mRNA expression was calculated using the 2-Δ ΔCt method with GAPDH as the internal control. The specificity of the PCR reaction was determined by the melting curve.

#### Protein extraction and Western blot analysis

The cells were washed twice with PBS. Lysate was used to lyse the cells. Samples were collected after lysing. After diluting the samples, protein concentrations were determined with the help of a kit. Based on the measured total protein concentration, a moderate amount of protein (30 μg) from the lysate was separated by 10% SDS-PAGE. Take out the gel, cut the target strip according to Marker, and use PVDF membrane and filter paper of the same size as the PAGE gel for membrane transfer. (Millipore, US). Subsequently, the primary antibodies was incubated and blocked with 5% skim milk: Anti-TGF-β1 antibody ( 1:1000), Anti-TGF-β3 antibody (1:500), Anti-CREB antibody (1:500), Anti-CollagenI antibody (1:1000), Anti-CollagenIII antibody (1:5000), rabbit antibody GAPDH (1:2500). After the primary antibody was incubated, the membrane was washed with 0.1% TBST for 3 times. The corresponding HRP was used to label the secondary antibodies, which were incubated at room temperature for 2 h. The LiCor Odyssey Imager(LI‐COR Biosciences, Lincoln, NE, USALICOR Biosciences, Inc., Lincoln, NE) was used to visualise and acquire images of proteins.

### Statistical analysis

The microscopic grading of the tendon healing process was performed by a K-W-rank sum test, and all other data were expressed as mean ± standard deviation (SD). The comparison between the two groups was performed using a t-test. Multiple comparisons were analyzed by one-way ANOVA followed by Fisher's test. *p* < 0.05 was regarded as statistically significant.

## Results

### Gait analysis

**Fig. 2:**
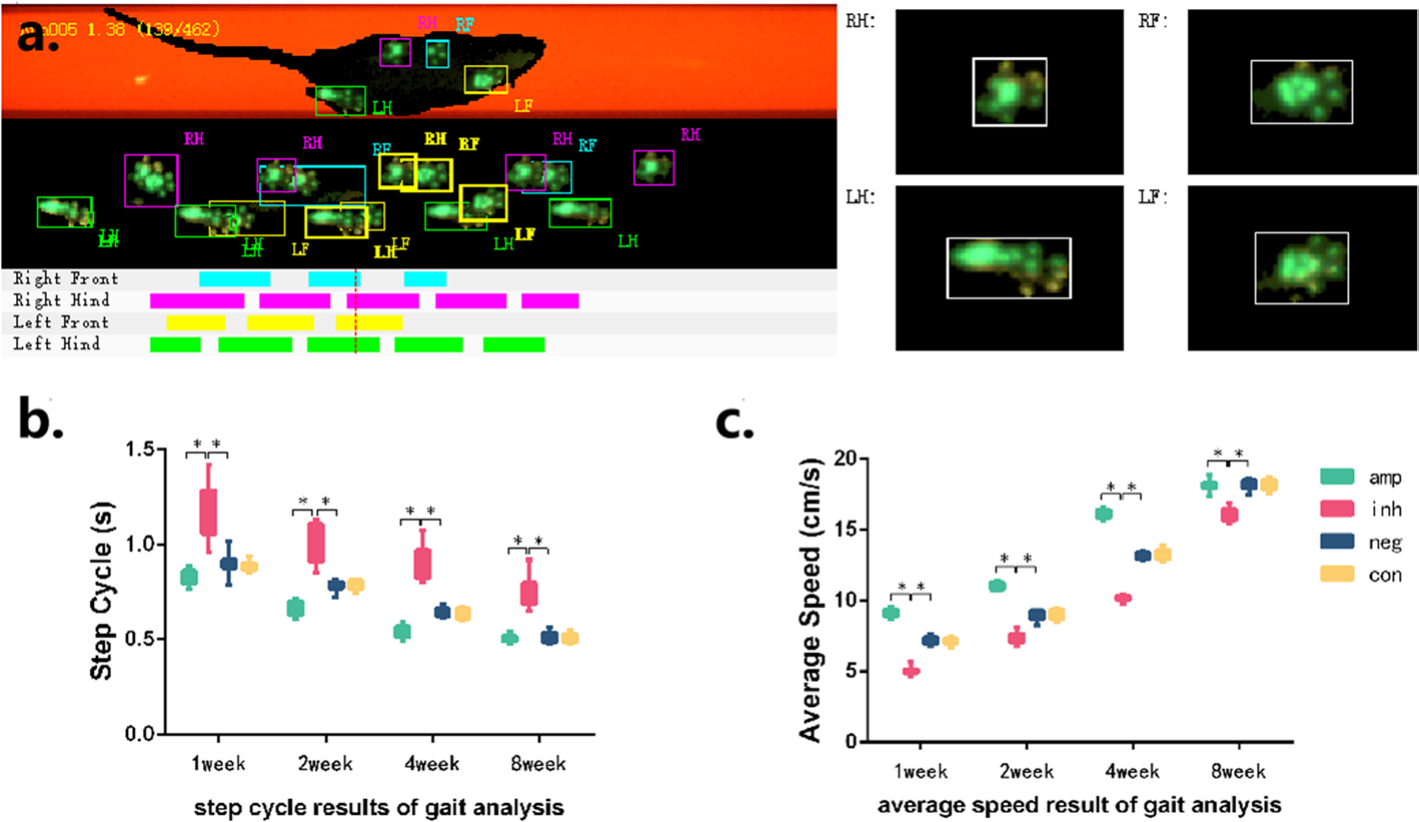
CatWalk gait analysis results: **a** Pattern diagram of gait analysis; **b** Result of step cycle. The result showed that the step cycle in the inhibition group was significantly longer than the other group at all study time points; **c** Result of average speed. The result showed that the average speed was significantly lower than the other experimental groups at all study time points. “ amp ” means amplification group, “inh”means inhibition group, “neg” means negative group, “con” means control group. Data were analyzed by $\overline{\mathrm{x}}$ ± s (*n* = 10). *: signifificant difference, *P* < 0.05

Gait analysis was performed using the CatWalk platform at 1, 2, 4, and 8 weeks. The step cycle was gradually reduced with time, but the inhibition group was significantly longer than the other experimental groups at each time point (all *p* < 0.05). Compared with the negative group and the control group, the amplification group was significantly shorter at 1,2, and 4 weeks after the operation (all *p* < 0.05), but there was no significant difference at 8 weeks after surgery (*p* > 0.05). The average speed was the lowest at 1 week after operation, and increased gradually with the recovery time, but the inhibition group 's speed was lower than the other experimental groups at each time point (all *p* < 0.05). All the experimental groups had recovered to a high level at 8 weeks after the operation, and the amplification group had no significant difference compared with the negative group and the control group (*p* > 0.05). The results showed that the improvement of gait behaviour in the amplification group was better than the inhibition group at each time point (Fig. 2).

### Gross observation

**Fig. 3:**
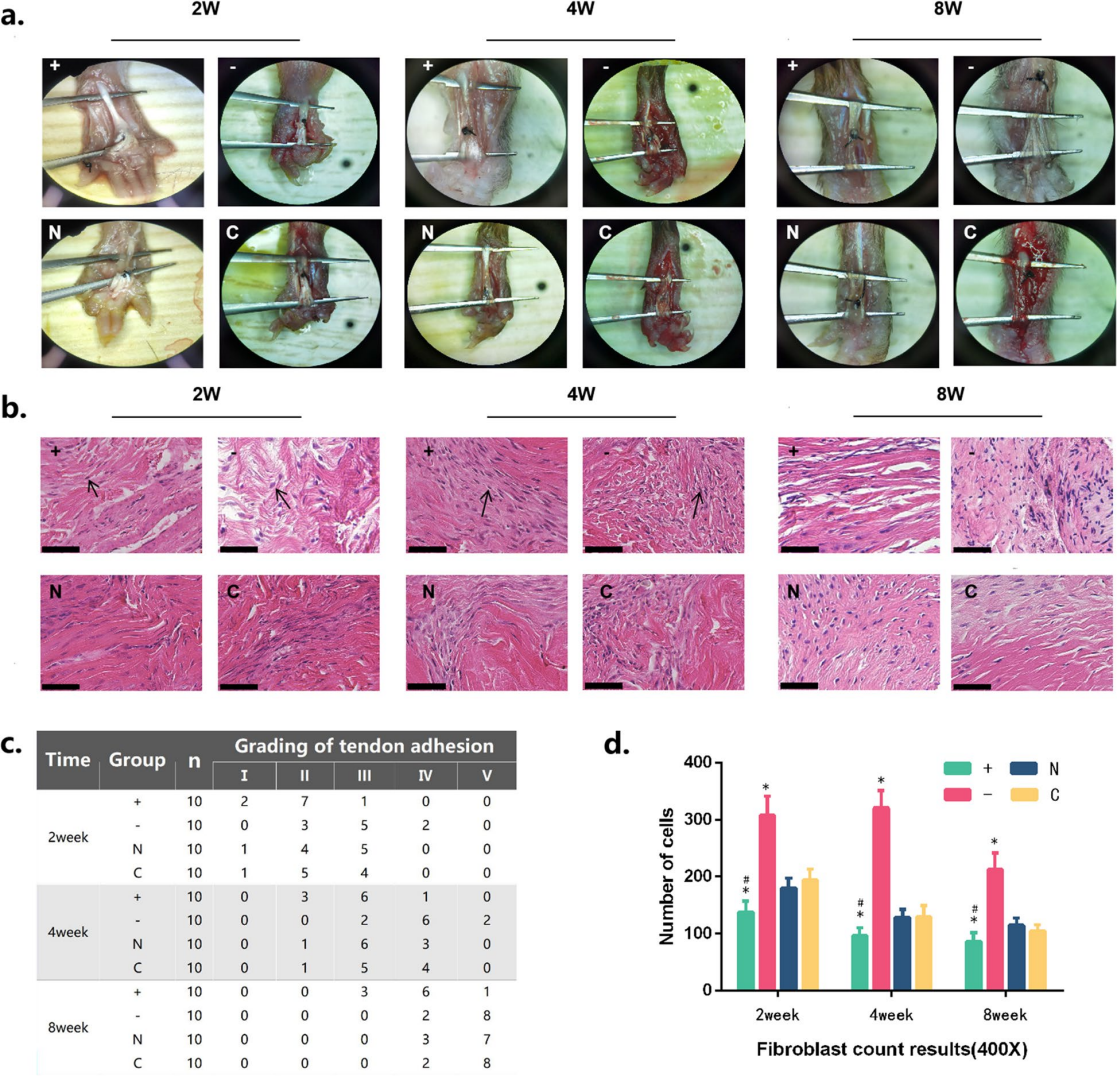
The gross healing and HE stain condition. **a** The general situation of adhesion around tendon. **b** HE staining microscope observation. The black arrow points to the fibroblasts. **c** Statistical results of tendon adhesion grading. The result showed that the amplification group have less adhesion than the other groups. **d** HE staining section fibroblast cell count. The result showed that the number of cells in amplification group was significantly lower than the other group, and the inhibition group was significantly higher than the other groups.“ + ” means amplification group, “-”means inhibition group, “N” means negative group, “C” means control group. scale bars: 50 um, *: compared with the negative and control group, *p* < 0.05, #: compared with the inhibition group, *P* < 0.05

By grading the tendon adhesion, it observed that the degree of adhesion in each experimental group was gradually aggravated with the extension of the study time, and almost all tendons reached extensive adhesion at 8 weeks after the operation. However, compared with the negative group and control group, the degree of adhesion in the amplification group was lower. It was significant at 2 weeks and 4 weeks (*p* < 0.05). The degree of adhesion in the inhibition group was greater than that in the negative group and control group. It was significant at 2 weeks and 4 weeks (*p* < 0.05). In the inhibition group, extensive adhesions were formed 4 weeks after the operation, while there were still some tendons that did not form extensive adhesion at 8 weeks in the amplification group (Fig. 3a, c).

### Histological observation

HE staining sections of the tendon specimens were obtained for cell count. The results showed that the number of cells in each experimental group increased at first and then decreased with the extension of the study time. The numbers of cells in the amplification group, negative group and control group were the highest at 2 weeks. There was no significant increase at 4 weeks in the inhibition group, but the number of cells at 4 weeks was still higher than at 2 weeks. The number of cells in the amplification group was lower than the inhibition group at each time point (all *p* < 0.05), and lower than the negative group and the control group (all *p* < 0.05). The inhibition group was higher than the negative and control groups (all *p* < 0.05), and was still significantly higher than the rest of the experimental group at 8 weeks after the operation (Fig. 3b, d).

### Immunohistochemistry

**Fig. 4:**
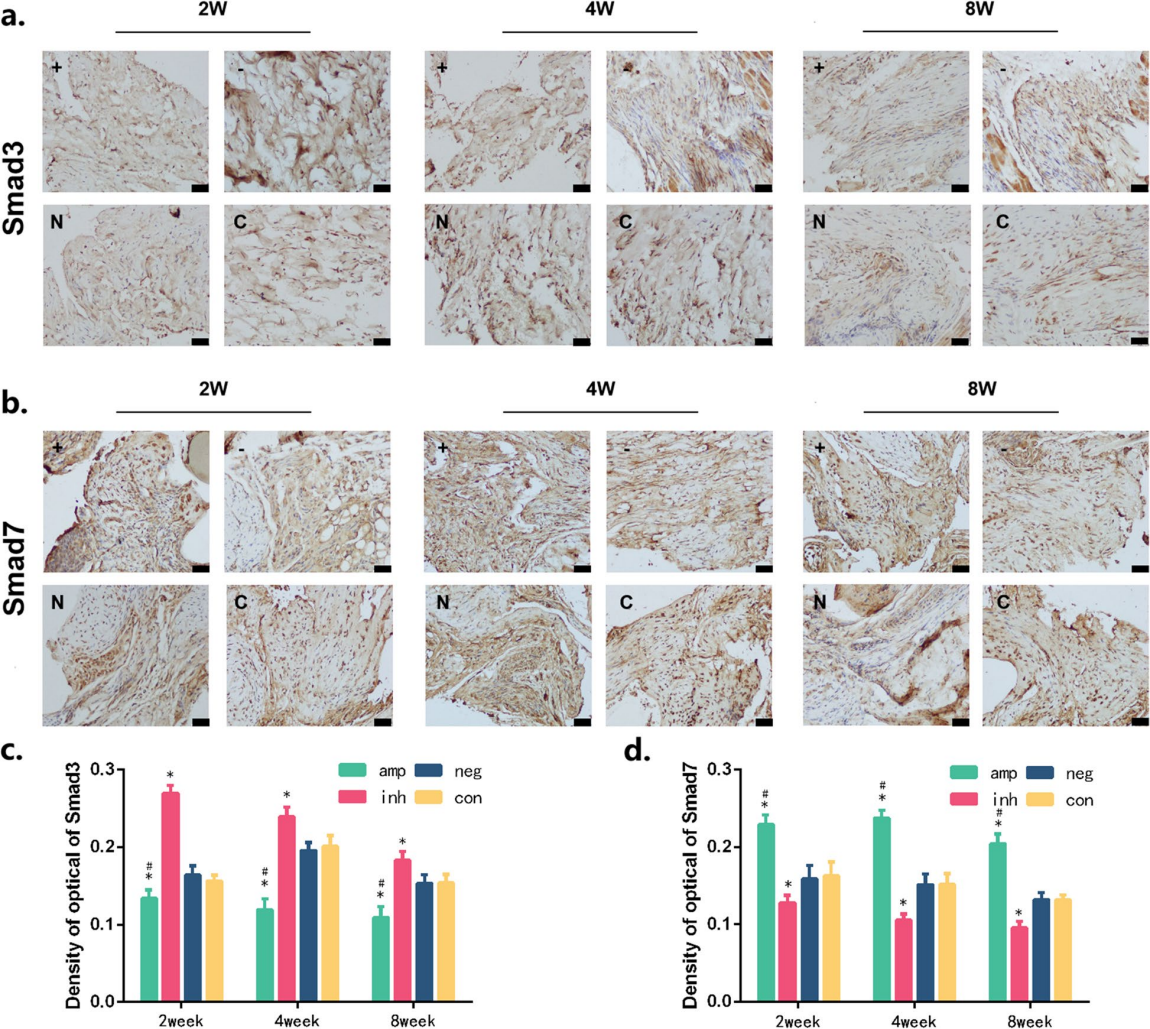
The immunohistochemical results and quantitation of Smad3 and Smad7. **a**, **c** Results and quantitation of Smad3. The result showed that the amplification group expressed less Smad3, and the inhibition group expressed more. **b**, **d** Results and quantitation of Smad7. The result showed that the amplification group expressed more Smad7, and the inhibition group expressed less. “ + ” means amplification group, “-”means inhibition group, “N” means negative group, “C” means control group. scale bars: 200 um, *:compared with the negative and control group, *P* < 0.05, #: group compared with the inhibition group, *P* < 0.05

**Fig. 5:**
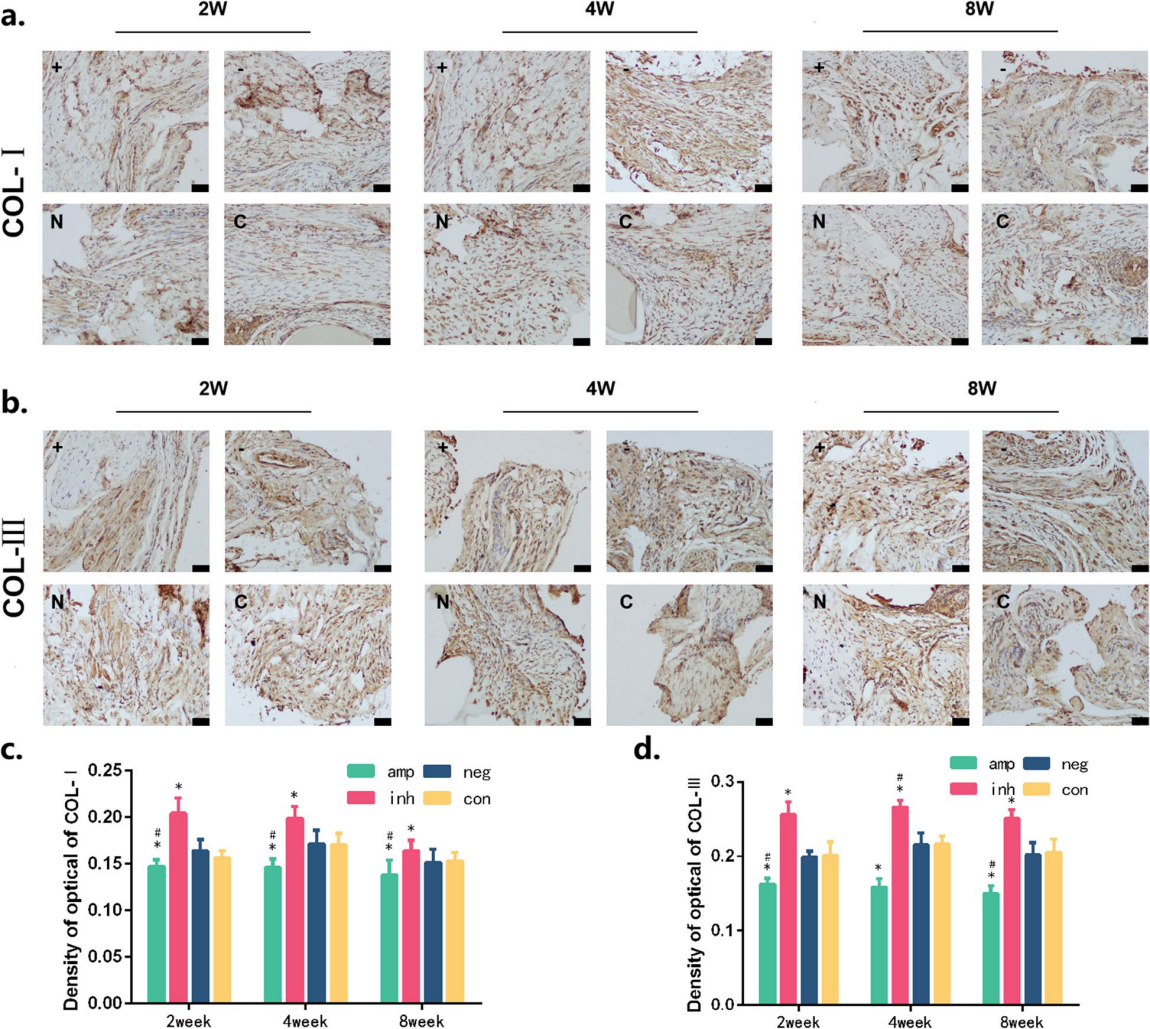
The immunohistochemical results and quantitation of COL-I and COL-III. **a**, **c** Results and quantitation of COL-I. The result showed that the amplification group expressed less COL-I, and the inhibition group expressed more. **b**, **d** Results and quantitation of COL-III. The result showed that the amplification group expressed less COL-III, and the inhibition group expressed more. “ + ” means amplification group, “-”means inhibition group, “N” means negative group, “C” means control group. scale bars: 200 um, *:compared with the negative and control group, *P* < 0.05, #: group compared with the inhibition group, *P* < 0.05

The tendon specimens from each group were stained by immunohistochemistry. The results showed that the protein expression of TGF-β3 in each experimental group showed a trend of increasing at first and then decreasing gradually, and the amplification group was significantly higher than the other experimental groups at all time points, while the inhibition group was significantly lower than the other experimental groups at all time points. The inhibition group, negative group and control group decreased significantly at 4 weeks compared with 2 weeks after the operation, however, there was no significant decrease in the amplification group at 4 weeks after the operation compared with 2 weeks. Though there was a significant decrease 8 weeks after the operation compared with 4 weeks, it was still significantly higher than the other experimental groups at the same time point. The protein expression of CREB-1 increased at first and then decreased. The protein expression of CREB-1 decreased significantly at 4 weeks after the operation compared to 2 weeks in the inhibition, negative, and control groups, but not in the amplification group. There was a significant reduction at 8 weeks after the operation compared with 4 weeks after the operation in all experimental groups. The expression of Smad3 in the amplification group was significantly lower than the inhibition group at all time points (all *p* < 0.05), and significantly lower than the negative group and control group (all *p* < 0.05). The inhibition group was significantly higher than the negative and control groups at all time points (*p* < 0.05). Smad3 protein expression reached its peak in all treatment groups two weeks after the operation. While the protein expression of Smad7 showed the opposite trend to that of Smad3 at each time point. The expression level of the amplification group was significantly higher than the inhibition group at all time points (all *p* < 0.05), and significantly higher than the negative group and control group (all *p* < 0.05). At all time points, the inhibition group was significantly lower than the negative and control groups (all *p* < 0.05). The amplification group reached its highest expression 4 weeks after the surgery. But the inhibition group had a downward trend at 4 weeks after the operation compared with 2 weeks. The expression of COL-I/III in the amplification group was significantly lower than the inhibition group (all *p* < 0.05), and significantly lower than the negative group and control group (all *p* < 0.05). The inhibition group was higher than the negative and control groups at all time points, and decreased gradually after reaching the highest level at 2 or 4 weeks after the operation. but the inhibition group was still significantly higher than other intervention groups at 8 weeks (Figs. 4 and 5).

### Collagen staining

**Fig. 6:**
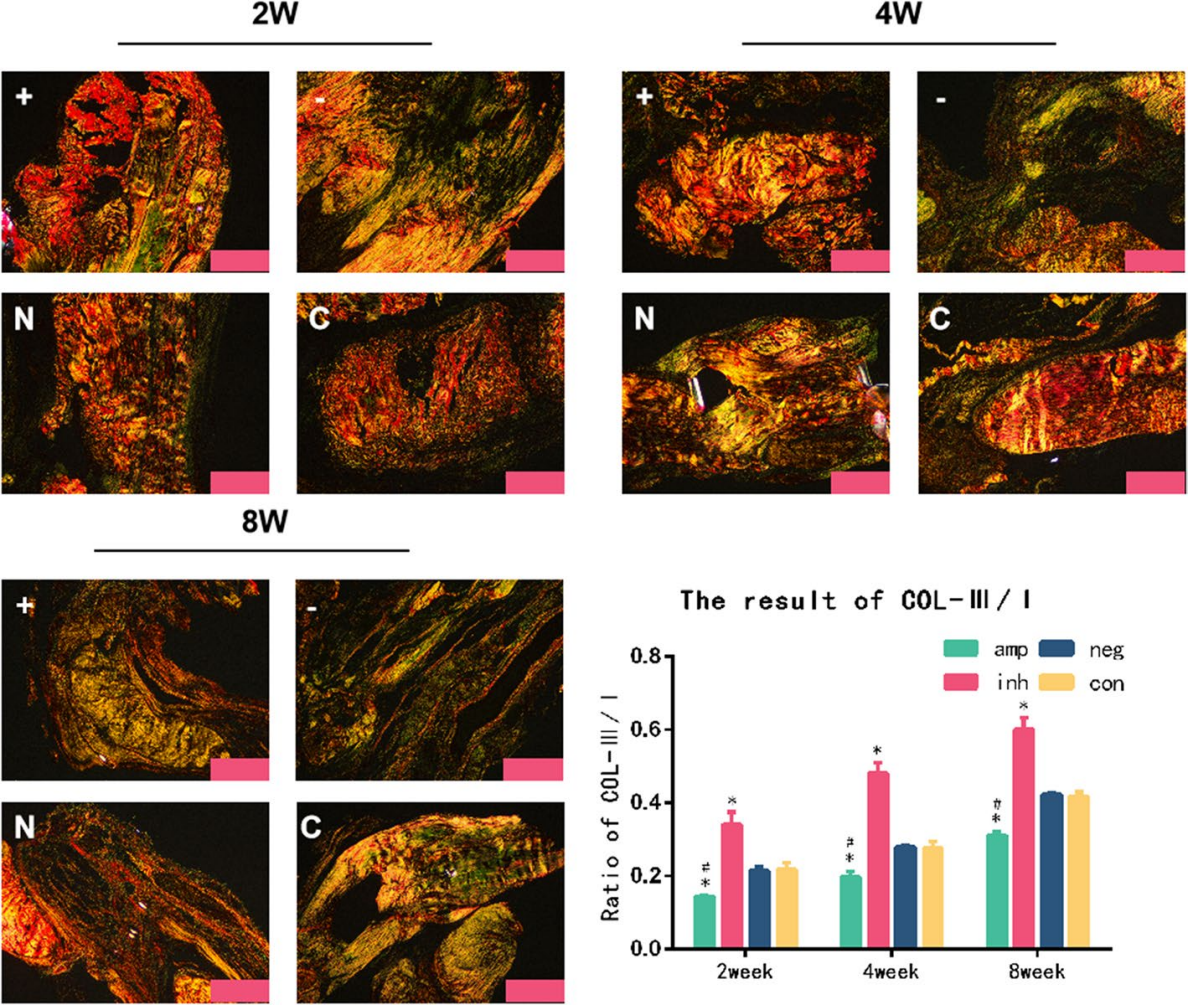
The result of Sirius red staining. (× 100) The result showed that the proportion of COL-III to COL-I in the amplification group was lower than the other groups, and the inhibition group was higher than the other groups.“ + ” means amplification group, “-”means inhibition group, “N” means negative group, “C” means control group. scale bars: 400 um, *:compared with the negative and control group, *P* < 0.05, #: group compared with the inhibition group, *P* < 0.05

The obtained tendon specimens were stained with Sirius red. The results indicated that the expression of COL-III in tendon tissue increased gradually with tendon healing. Moreover, at different time points, it can be found that the expression of COL-III in the amplification group is the least, while the expression of COL-III in the inhibition group is the most. The analysis of the proportion of COL-III to COL-I showed that the ratio in the amplification group was significantly lower than that in the other experimental groups (all *p* < 0.05), and the ratio in the inhibition group was significantly higher than that in other experimental groups (all *p* < 0.05) (Fig. 6).

### Cellular immunohistochemistry of TGF-β3, CREB, Smad3/7, COL-I/III, RT-qPCR of TGF-β3, CREB-1

**Fig. 7:**
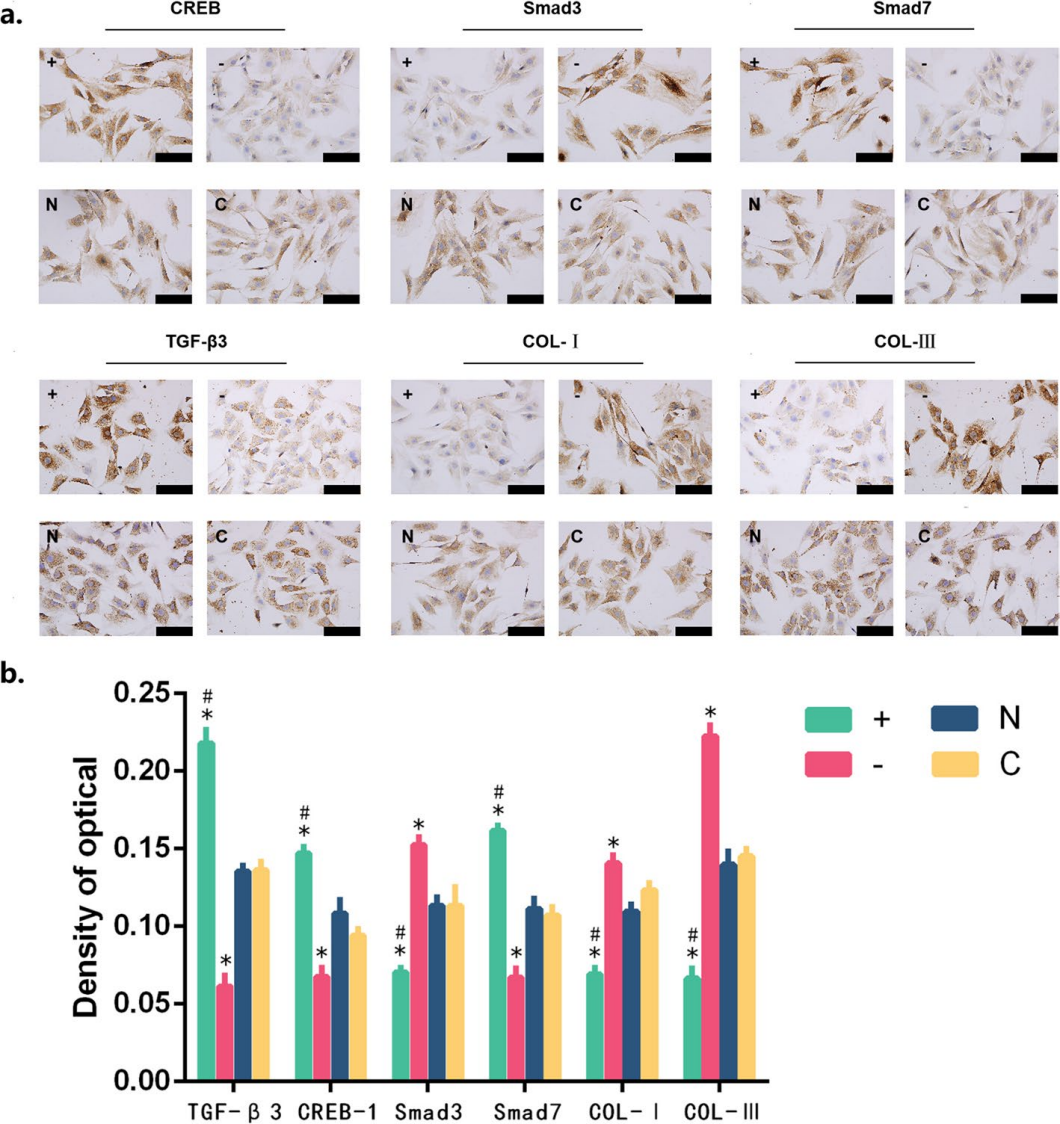
Result of Cellular immunohistochemistry. The result showed that the CREB, TGF-β3 and Smad7 expressed a high level in the amplification group, and the Smad3, COL-I and COL-III highly expressed in the inhibition group. The result of RT-qPCR are presented in Supplementary material. (Supplement Fig. 1) “ + ” means amplification group, “-”means inhibition group, “N” means negative group, “C” means control group. scale bars: 50 um, *: compared with the negative and control group, *P* < 0.05, #: compared with the inhibition group, *P* < 0.05

Immunohistochemistry was performed on the transfected tendon stem cells. The results showed that the amplification group had lower Smad3 and COL-I/III expression than the inhibition group, negative group, and control group (all *p* < 0.05), while the inhibition group had higher expression than other groups (all *p* < 0.05). And the expression of CREB, TGF-β3 and Smad7 were contrary. They were significantly higher in the amplification group than in the other experimental groups (all *p* < 0.05) (Fig. 7). The qPCR results showed that the mRNA transcription levels of TGF-β3 and CREB-1 in the amplification group were significantly higher than the other experimental groups (all *p* < 0.05), while those in the inhibition group were lower than the others (all *p* < 0.05).

### WB of TGF-β3, CREB-1, TGF-β1, COL-I/III

**Fig. 8:**
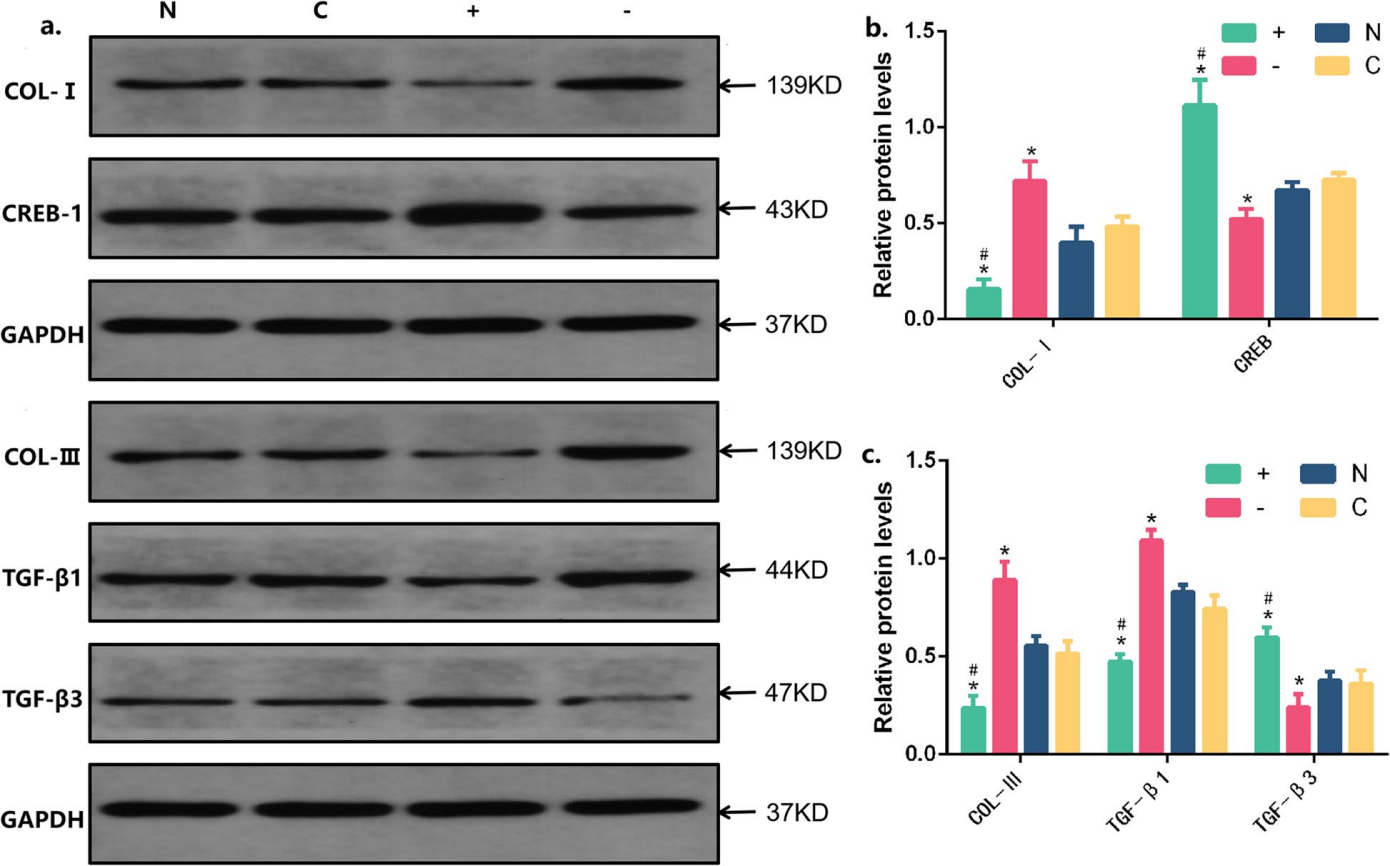
The WB result of protein expression: **a** Protein bands for TGF-β1/3, CREB-1, COL-I/III and GAPHD by western blotting assay. **b**, **c** Relative protein expressions of TGF-β1/3, CREB-1, COL-I/III in tendon stem cell by western blotting assay. Expression was normalized to GAPDH. The gel were cut before hybridizing with the antibody. The markers in the blots has been cropped and the original blots are presented in Supplementary material. (Supplement Fig. 2) The result showed that the CREB-1 and TGF-β3 highly expressed in the amplification group, and the TGF-β1, COL-I and COL-III highly expressed in the inhibition group. “ + ” means amplification group, “-”means inhibition group, “N” means negative group, “C” means control group. *: compared with the negative and control group, *P* < 0.05, #: compared with the inhibition group, *P* < 0.05

Western blot was used to detect the effect of the CREB-1 lentivirus on other cytokines after the transfection. The results showed that the protein expression levels of TGF-β3 and CREB-1 in the amplification group were significantly higher than the other groups (all *p* < 0.05), while the inhibition group was significantly lower than the other groups (all *p* < 0.05), but the expressions of TGF-β1, COL-I/III in the amplification group were significantly lower than the other groups (all *p* < 0.05). In contrast, the expression of TGF-β1, COL-I/III in the inhibition group was significantly higher than the other groups (all *p* < 0.05). The expression of CREB-1 and TGF-β3 was significantly lower than that of other groups in the inhibition group. (all *p* < 0.05) (Fig. 8).

## Discussion

The tendon is prone to adhesion in the healing process after injury. There are more patients with tendon adhesions caused by trauma or fixation. Without timely and effective treatment, the adhesion may lead to limited joint activity, affect joint function, and eventually lead to disability. In this study, we attempt to create a mouse tendon injury model in order to observe adhesion during the tendon healing process. The mechanism of the CREB-1 / TGF-β3 signaling pathway in the process was studied in detail.

Gait analysis, as a systematic, convenient and automatic method of animal behaviour evaluation, has been widely used in a large number of animal models of ischemic stroke, spinal cord injury, and Parkinson's disease [18, 19]. And we found that gait analysis has a correlation with the degree of tendon adhesion, which has good credibility for the evaluation of tendon healing [15]. Gait analysis was performed on animals after tendon injury in this study. We made cross-sectional comparisons between different intervention groups. The results showed that the step cycle in the amplification group was short and the average speed was fast at different observation time points, indicating that the animals had a good behavioural state, whereas the one in the inhibition group was poor. At the same time, we found that the gross observation of tendon showed a low degree of adhesion when the CREB-1 protein expression had been amplified. When the CREB-1 protein expression was inhibited, the tendon showed severe adhesion. These results indicated that regulating CREB-1 protein expression may affect the formation of tendon adhesion, and thus affect the behaviour of animals.

Due to the mediation of inflammatory factors after tendon injury, fibroblasts from peripheral tissue migrate to the injury area and produce scar tissue to repair the tendon [20]. But the scarring will form an adhesion with the surrounding tissue, and affect the function of the repaired tendon. TGF-β1 is an important growth factor for promoting tissue growth and healing [21], which mainly mediates the formation of fibrosis in tissue [22]. TGF-β1 protein expression has been found to be significantly increased in tendon injury [23], but the inflammatory induced by TGF-β1 can cause collagen tissue disorder and decrease the biomechanical properties of the tendon [24, 25]. Moreover, TGF-β1 mediates excessive scar formation, which will lead to increased adhesion between the scar and surrounding tissues. As an isomer of TGF-β1, TGF-β3 plays an antibody role of TGF-β1 and has the ability to inhibit scar formation and fibrosis [26–28]. High expression of TGF-β3 and low expression of TGF-β1 have been observed during scarless repair in embryos, while a large amount of TGF-β1 has been observed in adult scar wounds [29]. TGF-β3 protein expression was found to be significantly higher in the experimental group with mild tendon adhesion than in the group with severe tendon adhesion in this study. It is suggested that upregulation of TGF-β3 protein can reduce the adhesion in the tendon healing process, which is similar to the effect of adding exogenous TGF-β3 to promote tendon healing and reduce tendon adhesion [30]. TGF-β is primarily mediated by the Smads protein, and the TGF β / Smad signaling pathway plays an important role in fibrosis [31–33]. TGF-β3 upregulates mRNA and protein expression of Smad7 and downregulates Smad3 expression during the tendon healing process [30], thereby reducing scar formation and adhesion around the tendon. CREB-1, as a key downstream factor of TGF-β, may play a role in fibrosis processes in various tissues [13, 34, 35]. Studies have shown that CREB-1 overexpression can antagonize the fibrotic effects of TGF-β1 [36]. Another study reported that CREB-1 may be involved in the induction of Smad7 by TGF-β3 [37].

In this study, we observed the high protein expression of TGF-β1 and Smad3 in the experimental group with severe tendon adhesion, as well as the low protein expression of TGF-β3 and Smad7; In contrast, a higher protein expression of TGF-β3 and Smad7 levels were observed in the amplification group with less tendon adhesion, whereas the protein expression of TGF-β1 and Smad3 levels was lower.

CREB-1 is a key factor in the activation of TGF-β3, which can bind to the promoter and participate in the autocrine of TG-β3. Inhibition of CREB-1 could significantly reduce TGF-β3-induced Smad7 expression in vitro, while overexpression of CREB-1 enhanced the stimulatory effect of exogenous TGF-β3 on Smad7. These results suggest that CREB-1 may be involved in TGF-β3-induced Smad7 expression [37]. In our previous study, we found that TGF-β3 and CREB-1 are closely related to tendon healing, and may play a role in the anti-adhesion of tendon through TGF-β3 / CREB-1 signaling pathways [15]. This study further verified the relationship between CREB-1 and TGF-β3 in the tendon healing process. By upregulating CREB-1 protein expression, we observed that the protein expression of TGF-β3 was also increased. Contrastingly, the protein expression of TGF-β3 decreased after the protein expression of CREB-1 was inhibited. By regulating the protein level of CREB-1, it can affect the protein expression of TGF-β 3, and affect tendon healing.

Smad is a downstream protein of TGF-β. In the process of fibrosis, TGFβ-1 exerts its effects through downstream Smad2 and Smad3, but it is negatively regulated by Smad7 [31]. And TGF-β3 can induce Smad7 expression, indicating that the Smad3-mediated fibrosis process of TGF-β1 may be inhibited by the Samd7 pathway. In our experiment, high expression of Smad7 was also observed in the tendon injury group with high expression of TGF-β3 and low expression of Smad3. The low expression of TGF-β3 in the experimental group with heavy tendon adhesion was also associated with lower Smad7 and higher Smad3 expression. In the healing process of tendon injury, TGF-β3 may affect the adhesion of the tendon by regulating the downstream protein Smad7. Tendon tissue does not heal in a regenerative form after injury, but is replaced by scar tissue, which is different from tendon tissue in composition, structure and function [38]. Type I collagen is the most abundant component in the extracellular matrix of tendon tissue, and the proportion of type III collagen in tendon tissue increases during tendon scar repair [39]. The change in collagen composition in the extracellular matrix can lead to changes in the biomechanical properties of the tendon. Expression of type I and type III collagen mRNA was significantly increased in studies of Achilles tendinopathy [40, 41]. The protein expression of type I and III collagen was increased in the study of tendon injury, and the level of type III collagen remained high for a long time. The ratio of type III collagen to type I collagen was still higher than that of healthy tendons [39]. In our study, we also found that after tendon injury, indirect inhibition of TGF-β3 protein expression by inhibiting CREB-1 resulted in increased expression of type III / I collagen (type III / type I collagen ratio). This is consistent with the heavier adhesion with surrounding tissues after scar formation. And the lower expression of type III / I collagen (type III / I collagen ratio) in the experimental group with lighter adhesion further proves that CREB-1 may reduce tendon adhesion by regulating collagen expression in extracellular matrix via downstream Smad7, and via the signaling pathway of CREB-1 / TGF-β3.

Tendon cells are fibroblast-like cells located inside and around the collagen fibers. Tendon stem cells (TSCs) can be extracted from the tendons of humans, rabbits, rats and mice by cell culture technology [42], but the identification of TSCs is based on colony-forming unit analysis in cell culture. At present, TSCs cannot be observed in situ in tendons [38]. The major cells in tendon tissue are fibroblasts (tendon cells) [43]. In our animal experiments, only fibroblast-like cell proliferation was observed in the HE staining of tendon. Previous experiments have proved that tendon fibroblasts are closely related to the adhesion of tendons [14], and it has been proven that inhibiting fibroblast proliferation and collagen synthesis contributes to reducing the formation of tendon adhesion. When the CREB-1 protein overexpresses, we can observe that the number of cells in the tendon tissue is lower than in the other experimental group at the same time, which indicates that the local inflammation is lighter, and further explains the intrinsic reason for the lighter tendon adhesion. We further cultured tendon stem cells in vitro and transfected them with the CREB-1 amplification virus. We observed an increase in mRNA transcription and protein expression of CREB-1, and increased expression of TGF-β3 mRNA and Smad7 protein were also observed, while decreased expression of COL-I, COL-III and Smad3 were observed as well. On the contrary, inhibition of CREB-1 protein expression led to the opposite result. CREB-1 may be not just a cofactor in the TGF-β3-mediated transcription of Smad7. It may also directly mediate the increase of TGF-β3 protein expression, thereby promoting the expression of Smad7 downstream, inhibiting the TGF-β1 / Smad3 signaling pathway, and reducing the collagen deposition in the extracellular matrix.

In conclusion, our findings suggest that targeting the CREB-1 / TGF-β3 signaling pathway may be a novel strategy to prevent adhesion of tendon. There may be a pathway in tendon: CREB-1 promotes TGF-β3 mRNA transcription and increases TGF-β3 protein expression; TGF-β3 inhibits the activation and fibrotic effects of TGF-β1 / Smad3 signaling pathway via Smad7; COL-I / III protein deposition in the extracellular matrix (III / I collagen ratio) is reduced; and the fibrosis of tendon is also reduced, resulting in less adhesion in the tendon healing. However, how CREB-1 affects the detailed mechanism of TGF-β3 in tendon tissue is not clear, and further experiments are needed to study it.

## Supplementary Information

**Additional file 1:** **Supplement figure 1.** The result of RT-qPCR. The mRNA transcription levels of TGF-β3 and CREB-1 in the amplification group were significantly higher than the other experimental groups (all *p* < 0.05), while those in the inhibition group were lower than the others (all *p* < 0.05). **Supplement figure 2.** The membrane of WB. Each membrane has 5 groups. “ + ” means amplification group, “-”means inhibition group, “M”means marker, “N” means negative group, “ C ” means control group. During WB experiment, we cut the gel before hybridizing with the antibody. We have detailed descriptions in the experimental method and in the figure legend. The color of PVDF membrane we use is blue, we can see the shadow boundary of gel after membrane transfer. And we have also adjusted the image format to 300dpi as required.  

## Data Availability

The datasets generated for this study are available on request to the corresponding author.
