# Supplementary material for: Up-regulation of CREB-1 regulates tendon adhesion in the injury tendon healing through the CREB-1/TGF-β3 signaling pathway
Source: BMC Musculoskelet Disord. 2023 Apr 25;24:325. doi: 10.1186/s12891-023-06425-7 (PMC10127358; doi:10.1186/s12891-023-06425-7)
Supplement: Supplementary file 1 — Additional file 1: Supplement figure 1. The result of RT-qPCR. The mRNA transcription levels of TGF-β3 and CREB-1 in the amplification group were significantly higher than the other experimental groups (all p < 0.05), while those in the inhibition group were lower than the others (all p < 0.05). Supplement figure 2. The membrane of WB. Each membrane has 5 groups. “ + ” means amplification group, “-”means inhibition group, “M”means marker, “N” means negative group, “ C ” means control group. During WB experiment, we cut the gel before hybridizing with the antibody. We have detailed descriptions in the experimental method and in the figure legend. The color of PVDF membrane we use is blue, we can see the shadow boundary of gel after membrane transfer. And we have also adjusted the image format to 300dpi as required. [file 12891_2023_6425_MOESM1_ESM.pdf]

Supplementary material

1. The RT-qPCR result of TGF-β3, CREB-1

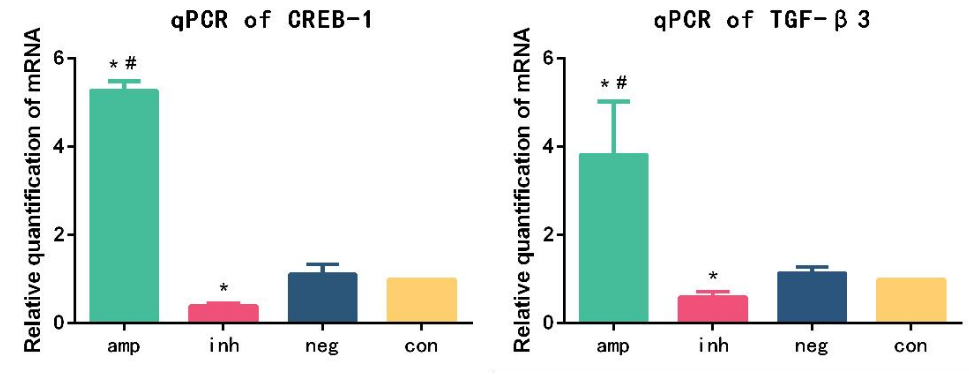

Supplement figure.1 The result of RT-qPCR. The mRNA transcription levels of TGF-β3 and CREB-1 in the amplification group were significantly higher than the other experimental groups (all $p < 0.05$), while those in the inhibition group were lower than the others (all $p < 0.05$).

2. The WB result of protein expression

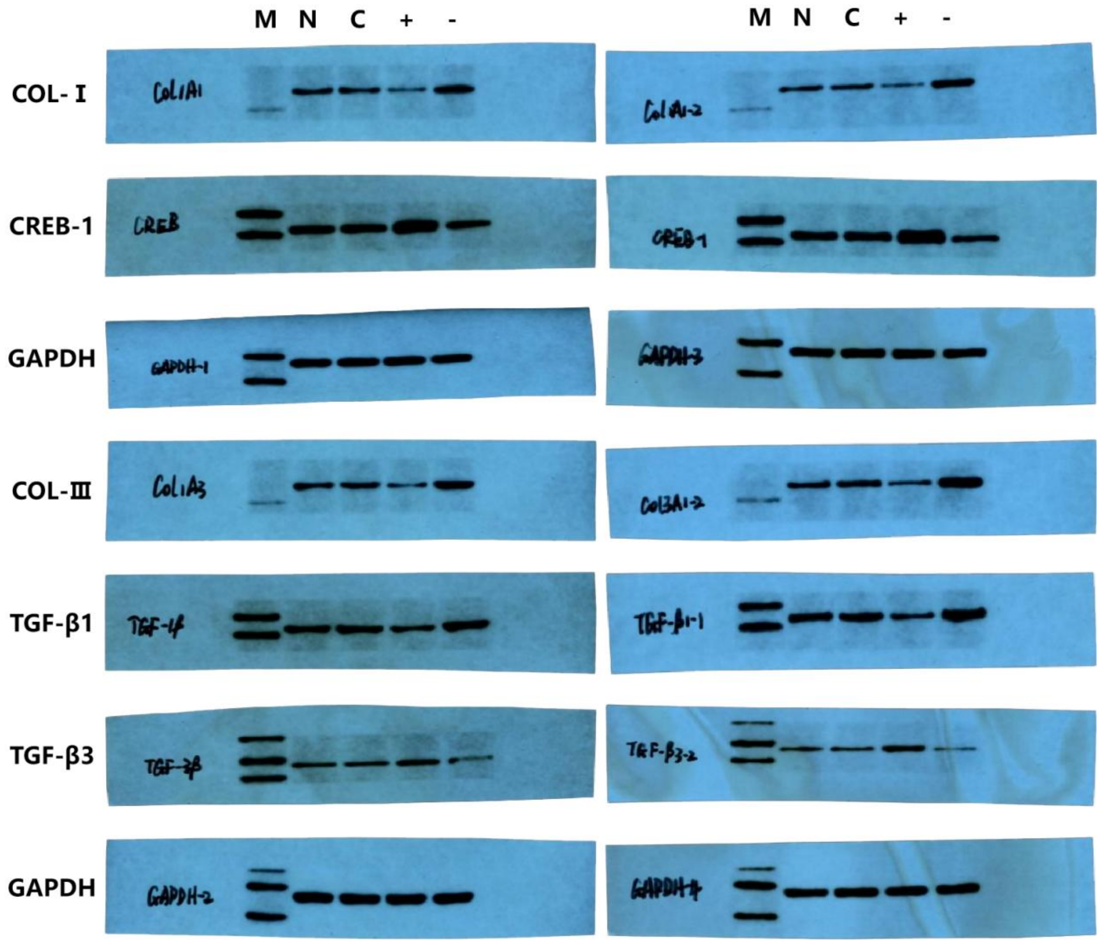

Supplement figure.2 The membrane of WB. Each membrane has 5 groups. “+” means

amplification group, “-”means inhibition group, “M”means marker, “N” means negative group, “C” means control group. During WB experiment, we cut the gel before hybridizing with the antibody. We have detailed descriptions in the experimental method and in the figure legend. The color of PVDF membrane we use is blue, we can see the shadow boundary of gel after membrane transfer. And we have also adjusted the image format to 300dpi as required.
